# Deterministic Single Cell Encapsulation in Asymmetric Microenvironments to Direct Cell Polarity

**DOI:** 10.1002/advs.202206014

**Published:** 2022-12-01

**Authors:** Ik Sung Cho, Prerak Gupta, Nima Mostafazadeh, Sing Wan Wong, Saiumamaheswari Saichellappa, Stephen Lenzini, Zhangli Peng, Jae‐Won Shin

**Affiliations:** ^1^ Department of Pharmacology and Regenerative Medicine University of Illinois at Chicago College of Medicine Chicago IL 60612 USA; ^2^ Department of Biomedical Engineering University of Illinois at Chicago Chicago IL 60607 USA

**Keywords:** cell polarity, microfluidics, hydrogels, microenvironments, cell encapsulation

## Abstract

Various signals in tissue microenvironments are often unevenly distributed around cells. Cellular responses to asymmetric cell‐matrix adhesion in a 3D space remain generally unclear and are to be studied at the single‐cell resolution. Here, the authors developed a droplet‐based microfluidic approach to manufacture a pure population of single cells in a microscale layer of compartmentalized 3D hydrogel matrices with a tunable spatial presentation of ligands at the subcellular level. Cells elongate with an asymmetric presentation of the integrin adhesion ligand Arg‐Gly‐Asp (RGD), while cells expand isotropically with a symmetric presentation of RGD. Membrane tension is higher on the side of single cells interacting with RGD than on the side without RGD. Finite element analysis shows that a non‐uniform isotropic cell volume expansion model is sufficient to recapitulate the experimental results. At a longer timescale, asymmetric ligand presentation commits mesenchymal stem cells to the osteogenic lineage. Cdc42 is an essential mediator of cell polarization and lineage specification in response to asymmetric cell‐matrix adhesion. This study highlights the utility of precisely controlling 3D ligand presentation around single cells to direct cell polarity for regenerative engineering and medicine.

## Graphical abstract

A droplet‐based microfluidic approach has been developed to encapsulate single cells at the interface between two distinct compartments in 3D Janus microgels. Using this approach, this study shows that mesenchymal stem cells elongate and polarize when one gel compartment contains a higher concentration of an integrin adhesion ligand than the other, resulting in enhanced osteogenic differentiation independently of gel mechanics.

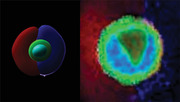

## 1 Introduction

The extracellular matrix in tissues consists of various biochemical, structural, and biophysical cues.^[^ ^1^ ^]^ At the microscale, cells often interact with local adhesive signals that are asymmetrically distributed. For example, bone marrow consists of microscale domains with distinct matrix compositions,^[^ ^2^ ^]^ and some stem cells and progenitors in the marrow are in contact with multiple domains in vivo.^[^ ^3^ ^]^ One important biological consequence of asymmetric cell‐matrix interactions is the establishment of cell polarity.^[^ ^4^ ^]^ Defects in cell polarity have broad implications in driving pathological processes, including developmental defects, aging, and cancer.^[^ ^5^ ^]^ The process of establishing mammalian cell polarity has been studied on 2D rigid cell culture substrates based on how protein complexes become spatially segregated to the discrete regions of a cell over time. Spatial segregation of protein complexes changes cytoskeletal structures that dictate cell morphology and functions^[^ ^6^, ^7^ ^]^ or alters the expression of regulatory factors that become partitioned into one of the daughter cells during division, leading to lineage specification.^[^ ^8^ ^]^

To study cellular responses to asymmetric cell‐matrix adhesion at the single‐cell resolution, it is important to spatially control the presentation of external cues at the microscale. Micropatterning was previously used to control the spatial presentation of matrix molecules underneath single cells and hence, has served as a predominant approach to studying cell polarity on 2D substrates. The anisotropic distribution of cell adhesion not only polarizes cytoskeleton organization^[^ ^9^, ^10^ ^]^ but also drives cell migration^[^ ^11^ ^]^ and asymmetric segregation of DNA during cell division.^[^ ^12^ ^]^ In addition, micro‐to‐nanoscale variations in mechanics^[^ ^13^ ^]^ and topography^[^ ^14^ ^]^ of 2D substrates are known to result in polarized cell shape, directed migration, and even lineage specification. The maintenance of cell polarity upon adhesion on 2D substrates via integrins not only requires classical cell polarity regulators, such as the small GTPase Cdc42^[^ ^15^ ^]^ but also involves the mechanotransduction machinery, including actomyosin contractility,^[^ ^6^ ^]^ nuclear lamins,^[^ ^16^ ^]^ and mechanosensitive transcription factors.^[^ ^17^ ^]^

Cellular responses to asymmetric cell‐matrix interactions in a 3D space remain poorly understood and controlled due to a lack of methods to precisely tune material properties at the subcellular level in a 3D space. Previous studies show that cells can polarize and undergo spreading in degradable,^[^ ^18^ ^]^ fast stress relaxing,^[^ ^19^ ^]^ or plastic^[^ ^20^ ^]^ 3D hydrogels, where cells can overcome spatial confinement. However, cell polarity is likely established randomly in this context, since there is no directional signal present within hydrogels. The prospect of developing single‐cell encapsulation approaches is attractive because they enable the precision control of cell‐matrix interactions at the single‐cell level. Previous studies encapsulated single cells in microwells fabricated within a bulk 3D hydrogel and showed the importance of niche geometry on stem cell shape, mechanotransduction, and differentiation.^[^ ^21^ ^]^ By using droplet‐based microfluidics, it is possible to encapsulate single cells in microscale hydrogels (microgels) with lower material‐to‐cell volume ratios while tuning gel properties,^[^ ^22^ ^]^ which could reveal biological phenotypes that were not previously observed with bulk hydrogels. For example, mesenchymal stem cells (MSCs) undergo adipogenic differentiation in an alginate‐Arg‐Gly‐Asp (RGD) hydrogel with low elasticity.^[^ ^23^ ^]^ However, when the amount of the alginate‐RGD gel per cell is reduced while keeping the same gel elasticity, MSCs undergo osteogenic differentiation, which was shown to be associated with increased isotropic cell volume expansion and membrane tension in the presence of RGD.^[^ ^24^ ^]^ However, existing single‐cell encapsulation approaches yield conformal gel coatings around single cells with uniform properties. To control matrix‐directed single‐cell polarity in a 3D space, it will be important to compartmentalize gel coatings where the properties of each compartment can be independently controlled. While compartmentalized microgels were fabricated to pattern different cell populations^[^ ^25^ ^]^ or to pair cells,^[^ ^26^ ^]^ there have been no reports that demonstrate single‐cell encapsulation in patterned gel coatings and the utility of this approach in controlling single‐cell polarity.

Here, we present a method where a pure population of single cells encapsulated in compartmentalized microgel coatings can be generated. By leveraging this method, we show that it is possible to precisely control the polarity of cell‐matrix interactions by tuning the spatial presentation of cell adhesion ligands around single cells in a 3D space. We report the fundamental process where single cells become elongated in shape and undergo polarization of membrane tension when integrin ligands are asymmetrically distributed. This process is sufficient to commit single MSCs to the osteogenic lineage, suggesting the functional implication of 3D substrate‐directed single stem cell polarization in lineage specification.

## 2 Results

### 2.1 Deterministic Single Cell Encapsulation in Microgels with Tunable Spatial Presentation of Ligands

**Figure 1:**
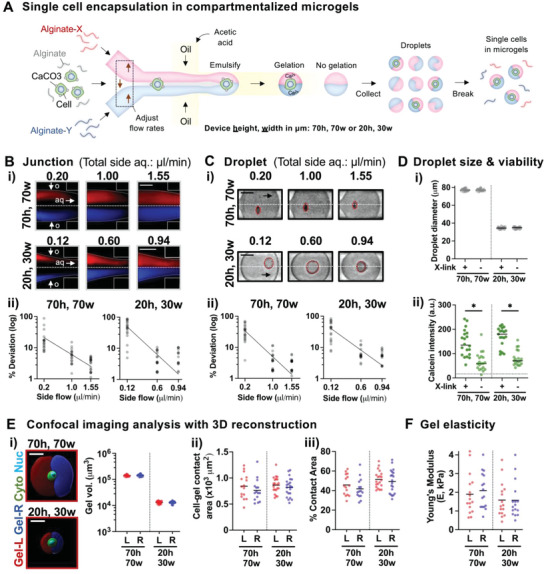
Encapsulation of single cells in compartmentalized microgels. A) Scheme illustrating the droplet microfluidic device to form conformal gel coatings with distinct compartments around single cells (see the Experimental Section). The device consists of three inlets for alginate‐based aqueous phases where the composition of each compartment can be independently tuned from each other. CaCO_3_‐coated cells with unmodified alginate were added to the middle aqueous phase, while fluorescently labeled alginate was added to each side aqueous phase to visually distinguish different compartments. To focus cells in the middle part of the aqueous phase, the flow rate of the middle aqueous phase was progressively reduced, while that of the side aqueous phases was increased, keeping the total aqueous flow rate constant. The oil phase consists of fluorinated oil, surfactant, and acetic acid. After emulsification at the junction, the droplets that contain CaCO_3_‐coated cells undergo gelation due to Ca^2+^ release by acetic acid, while those that do not contain the cells do not undergo gelation. This results in a pure population of cells in compartmentalized gel coatings. The device with height and width (in µm) = 70h, 70w or 20h, 30w was used to form large and small gel coatings, respectively. B) The aqueous phases maintain laminar flow at the junction. i) Representative images showing the compartmentalized aqueous (aq) phases with varied flow rates as they contact the oil (o) phase. Each side aqueous phase consists of alginate‐rhodamine (red) or alginate‐CF^TM^ 350 (blue). Scale bar = 25 µm. ii) Location of cells with respect to the midline of the aqueous phase at the junction. *n* = 15–20 cells for each group. The data were fitted to an exponential decay equation *Y* = *Y*_0_^.^e^−kX^ with *Y*_0_ and half‐life (ln2/k) for 70h, 70w = 29.0%, 0.44 µl min^−1^; 20h, 30w = 81.4%, 0.16 µl min^−1^. C) Cells remain closer to the midline with lower side aqueous flow rates after droplet formation in the absence of gel crosslinking. i) Representative images showing droplets containing single cells in the channel after emulsification. Scale bar for 70h, 70w = 20 µm; 20h, 30w = 10 µm. ii) Location of cells with respect to the midline of the droplets in the channel. *n* = 16–20 cells for each group. The data were fitted to an exponential decay equation *Y* = *Y*_0_^.^e^−kX^ with *Y*_0_ and half‐life (ln2/k) for 70h, 70w = 55.3%, 0.29 µl min^−1^; 20h, 30w = 59.9%, 0.21 µl min^−1^. D) Gel crosslinking (X‐link) is necessary upon emulsification to maintain cell viability in droplets. i) Droplet size after collection. *n* = 20 cells for each group. ii) Mean calcein intensity of single CaCO_3_‐coated cells in droplets after collection in the presence or absence of acetic acid in the oil phase. *n* = 20 cells. *p < 0.0001 via Welch's *t*‐test. The dotted horizontal line indicates a background mean intensity level. E) Confocal imaging analysis of single cells in compartmentalized gel coatings. i) (Left) Representative 3D‐reconstructed confocal images show single MSCs encapsulated in between two gel compartments ≈8 h after encapsulation. Red: alginate‐rhodamine (Gel‐Left), Blue: alginate‐CF^TM^ 647 (Gel‐Right), Green: cytoplasm (calcein), Cyan: nucleus (Hoechst). Scale bar = 20 µm. (Right) The volume of each gel compartment per gel‐coated cell. Left: alginate‐rhodamine, Right: alginate‐CF^TM^ 647. *n* = 18 for 70h, 70w, *n* = 29 for 20h, 30w. ii) Contact area and iii) percentage of contact area relative to total cell surface area between the cell and each gel compartment. *n* = 15 for 70h, 70w, *n* = 21 for 20 h, 30w. F) Young's modulus (*E*) of each gel compartment remains constant at ≈2 kPa as measured by AFM. *n* = 16 for each group.

To control the spatial presentation of ligands around single cells in a 3D space, we developed a droplet‐based microfluidic approach to manufacture a pure population of single cells that are placed between two distinct compartments (“Janus”) in microgels. To achieve this goal, a microfluidic device was designed with three distinct input channels (two side channels and one middle channel) for aqueous phases that merge at the junction prior to droplet formation (**Figure** **1**A and Figure S1A, Supporting Information). The devices with two different dimensions (height, width in µm = 70 h, 70w or 20 h, 30w; Figure S1B, Supporting Information) were fabricated to test whether distinct compartments can be formed within single cell‐encapsulating microgels with varied sizes. High‐viscosity alginate (LF200, ≈240 kDa, viscosity = 200–400 mPa^.^s, 1% w/v) was chosen as a base material for all of the aqueous phases to minimize the possibility of mixing. A small fraction of fluorescently conjugated alginate was added as a model gel‐bound ligand to visualize each aqueous phase in the side channels. The aqueous phase maintains laminar flow not only at the junction but also when the oil phase is about to pinch off the aqueous phase into droplets (Figure 1B‐i). To place single cells between two compartments, cells were added to the middle channel. MSCs were chosen as a model cell type to study cell‐matrix interactions.^[^ ^18^, ^22^, ^23^, ^27^, ^28^, ^29^, ^30^ ^]^ Clonally derived murine D1 MSCs (≈15.6 µm in diameter or ≈2000 µm^3^ in total cell volume) were used since they are known to show less cell‐to‐cell heterogeneity compared to primary cells.^[^ ^23^, ^28^, ^29^, ^30^ ^]^ The flow rate of the middle aqueous channel was then progressively reduced, and that of the side channels was increased, while the total aqueous flow rate was kept constant. This process not only focuses cells in the middle of the aqueous phase at the junction but also allows cells to contact the side aqueous phases (Figure 1B‐ii). Cells remain localized in the midline of the droplets even after droplet formation without alginate crosslinking (Figure 1C). However, in order to purify single cells in microgels, we coated cells with CaCO_3_ nanoparticles and washed out the excess so that the gelation of alginate can occur only when droplets contain cells.^[^ ^22^, ^24^ ^]^ The cell concentration was kept at ≈15 million per ml in the middle aqueous phase to maximize the number of purified cells in microgels while keeping the average of one cell per microgel (≈90% of the total population; Figure S1C, Supporting Information). While the size of single cell‐containing droplets depends on the device dimensions (Figure 1D‐i), immediate crosslinking of alginate upon droplet formation by adding acetic acid in the oil phase to release Ca^2+^ from CaCO_3_ on the cell surface is required to maintain cell viability as indicated by calcein staining (Figure 1D‐ii). To this end, this method results in a purified population of single cells localized in the middle of Janus microgels with varied sizes (Figure 1E‐i), and each cell contacts with both compartments to an equal extent (Figure 1E(ii,iii)). In addition, Young's modulus (*E*) of each gel compartment remains the same at ≈2 kPa (Figure 1F) and is tunable down to ≈0.5 kPa by lowering the CaCO_3_ concentration (Figure S1D, Supporting Information), the values that are exhibited by natural soft tissues.^[^ ^31^ ^]^ Thus, this method can be used to present hydrogel matrix‐bound ligands to single cells either symmetrically or asymmetrically independently of the base polymer, gel size, and gel elasticity.

### 2.2 Controlling the Spatial Presentation of Integrin Ligands around Single Cells in a 3D Space

**Figure 2:**
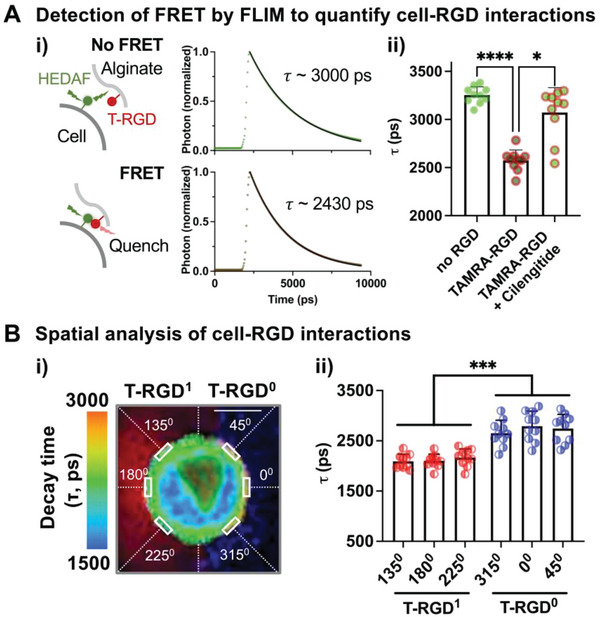
Quantification of single cell‐integrin ligand interactions in compartmentalized microgels. A) Characterization of the fluorescence lifetime imaging microscopy (FLIM) technique to detect Förster resonance energy transfer (FRET) as a result of cell‐ligand interactions. i) Prior to encapsulation, MSCs were labeled with the membrane dye 5‐hexadecanoylaminofluorescein (HEDAF), which serves as a donor. When HEDAF‐labeled cells interact with the tetramethylrhodamine (TAMRA)‐labeled integrin ligand Arg‐Gly‐Asp (T‐RGD) conjugated to alginate microgels (≈60 µM at 1% w/v), T‐RGD serves as an acceptor, leading to the reduction in donor fluorescence due to FRET. Thus, FRET results in a faster decay in the fluorescence lifetime (*τ*) of the donor as shown in representative graphs from FLIM. ii) Reduction in *τ* is specifically due to the molecular interaction between integrins and RGD. *τ* of the donor was measured after encapsulating HEDAF‐labelled MSCs in microgels (≈1.0 × 10^4^ µm^3^ in volume) with alginate‐rhodamine but without alginate‐RGD (no RGD) or microgels with T‐RGD. In one group, MSCs in T‐RGD microgels were treated with cilengitide (200 nM) for 2 h at 37 °C prior to FLIM. *n* = 10 cells. *p < 0.05, ****p < 0.0001. B) Subcellular analysis of *τ* in compartmentalized microgels with asymmetric RGD presentation. i) Representative image showing *τ* values across different regions (angles in counterclockwise directions from 0^0^) of the cell membrane in a microgel consisting of T‐RGD in one compartment (red, T‐RGD^1^) and no RGD but with CF^TM^ 350 in the other compartment (blue, T‐RGD^0^). Scale bar = 10 µm ii) *τ* values from MSCs in gels. *n* = 10 cells. ***p < 0.001. Individual *p* values were derived from Welch's ANOVA, followed by Dunnett's T3 multiple comparison test. All data are shown as mean ± SD.

We sought to leverage the method to understand cellular responses to asymmetric cell‐matrix adhesion at the single‐cell level in a 3D space. The RGD ligand was chosen as an integrin ligand due to its ability to interact with a number of integrins, including *α*_5_ *β*_1_ and *α*_v_ *β*_3_.^[^ ^32^ ^]^ To quantify the interaction between the cell membrane and RGD in the gel, we leveraged a Förster resonance energy transfer (FRET)‐based approach by labeling the cell membrane of MSCs with a fluorescein‐based dye (donor) and conjugating the tetramethylrhodamine (TAMRA)‐labeled RGD (T‐RGD) peptide (acceptor) to alginate as described.^[^ ^33^ ^]^ Here, fluorescence lifetime imaging microscopy (FLIM) was used, since it enables FRET measurements independently of fluorophore concentrations^[^ ^34^ ^]^—with FRET, the donor emission becomes quenched, resulting in a decreased fluorescence lifetime (*τ*; **Figure** **2**A‐i). Encapsulating fluorescein‐labeled cells in microgels with T‐RGD decreases *τ*, while treatment with cilengitide, a soluble cyclic RGD peptide that competitively inhibits integrins,^[^ ^35^ ^]^ restores *τ* (Figure 2A‐ii). Thus, decreased *τ* is selectively due to integrin‐RGD interactions. Importantly, subcellular analysis shows that *τ* is decreased only on the side of single cells that are in contact with T‐RGD when added to one of the compartments in Janus microgels (Figure 2B). Thus, our approach can control the polarity of integrin‐ligand interactions in a 3D hydrogel matrix around single cells.

### 2.3 Accelerated Elongation of Single Cells by Asymmetric Presentation of Integrin Ligands

**Figure 3:**
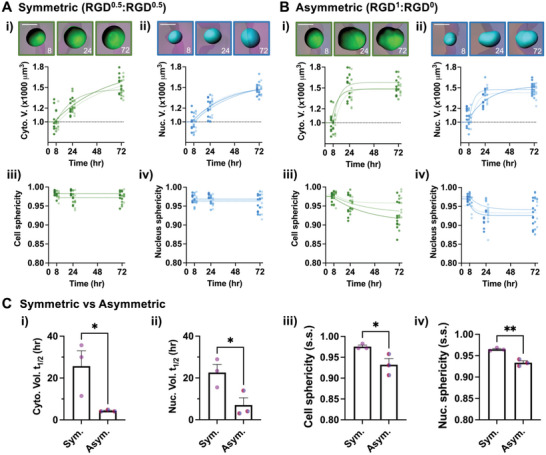
Spatial presentation of integrin ligands around single cells impacts cell volume expansion and shape. A) For symmetric RGD presentation, alginate conjugated with ≈60 µM RGD at 1% w/v (RGD^1^) was equally mixed with unmodified alginate (RGD^0^) and added to both compartments (≈30 µM RGD at 1% w/v; RGD^0.5^). B) For asymmetric RGD presentation, RGD^1^ was added to one compartment, and RGD^0^ was added to the other compartment. For both A) and B), the kinetics data with representative images (scale bar = 15 µm) are shown for i) cytoplasmic volume, ii) nuclear volume, iii) cell sphericity, and iv) nucleus sphericity. The volume data from each experiment were fitted to a one‐phase association equation starting from *t* = 8 h: *V* = *V*_0_ + (*V*_m_−*V*_0_) (1 – e^−kt^), where *V*_0_ = 1000 µm^3^. The sphericity data were fitted to a linear equation with slope = 0: S = S_0_, for symmetric RGD presentation, and an exponential decay equation starting from *t* = 8 h: *S* = *S*_p_ + (*S*_0_−*S*_p_) e^−kt^, for asymmetric RGD presentation. C) Comparison of the kinetics parameters between symmetric and asymmetric RGD presentations. The parameters include half‐maximum times (*t_1/2_*, in h) for i) cytoplasmic and ii) nuclear volume expansion and steady‐state (s.s.) sphericity of iii) cells and iv) nuclei. *n* = 3 independent experiments. *p < 0.05, **p < 0.01 via unpaired *t*‐test. All data are shown as mean ± SD.

To understand how the polarity of cell‐matrix interactions impacts single‐cell morphology and volume in a 3D space, single MSCs were encapsulated in microgels with either symmetric or asymmetric RGD presentation. A thinner gel (≈2.3 × 10^4^ µm^3^ in total volume or ≈10 µm in thickness, Figure 1E) was chosen to encapsulate single MSCs for downstream analyses. MSCs in the microgels were subsequently embedded in a collagen‐I gel at a low density (≈10 000 cells in 50 µl), labeled with calcein or Hoechst to visualize the cytoplasm and the nucleus, respectively, followed by confocal imaging analysis to evaluate their volume changes over time in a 3D space. Without RGD in microgels, the volume of the cytoplasm and nucleus was previously determined to be around 1000 µm^3^ each for mouse MSCs.^[^ ^24^ ^]^ With symmetric RGD presentation, MSCs expand in volume over 3 days by ≈1.5‐fold but remain spherical, suggesting isotropic expansion (**Figure** **3**A). Increasing the total RGD concentration by 2‐fold in both compartments does not impact cell volume expansion kinetics or sphericity (Figure S2A, Supporting Information). In contrast, when RGD is asymmetrically distributed, MSCs expand in volume faster, while both cell sphericity and nucleus sphericity decrease over time (Figure 3B,C), indicating cell elongation. The gel volume of both compartments remains unchanged over 3 days in culture (Figure S2B,C, Supporting Information). Using an intermediate ligand ratio between the two compartments (left: right = 0.75: 0.25) shows the progressive acceleration in cell volume expansion and decrease in sphericity from symmetric to asymmetric RGD presentation (Figure S2D, Supporting Information). Consistent with previous studies,^[^ ^22^, ^24^, ^31^ ^]^ most mouse MSCs in the high molecular weight alginate gel with asymmetric RGD presentation do not proliferate over 3 days in culture, as confirmed by a low level of 5‐ethynyl‐2′deoxyuridine (EdU) incorporation into the nucleus for 3 days, as opposed to most MSCs in collagen‐I gel, which incorporates EdU within 1 day in culture (Figure S2E, Supporting Information). Lowering gel elasticity to ≈0.5 kPa does not increase the proliferation of encapsulated MSCs (Figure S2E, Supporting Information). Together, polarized cell‐matrix interactions increase the probability of single cells undergoing volume expansion by elongation.

### 2.4 Polarized Membrane Tension in Single Cells by Asymmetric Presentation of Integrin Ligands

**Figure 4:**
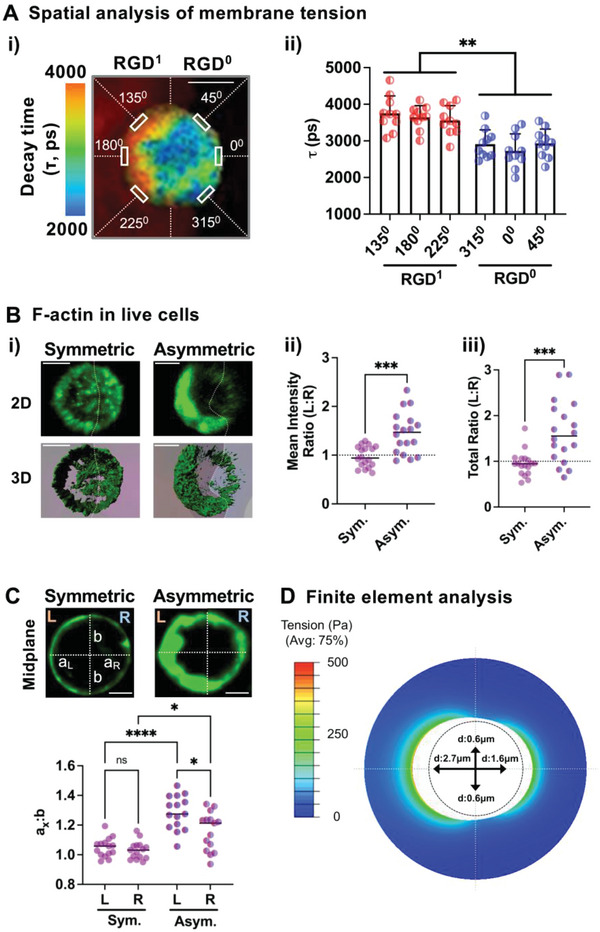
Asymmetric cell‐matrix adhesion leads to polarized membrane tension in single cells. A) Measurement of membrane tension at 1 day after encapsulation of MSCs in compartmentalized microgels. Fluorescence lifetime imaging microscopy (FLIM) was used to evaluate the decay lifetime (*τ*) of the lipid tension reporter on the cell membrane (Figure S3A, Supporting Information). i) Representative image showing *τ* across different regions (angles in counterclockwise directions from 0^0^) of the cell membrane in a microgel consisting of ≈60 µM 1% w/v alginate‐RGD (RGD^1^) on the left side and unmodified RGD (RGD^0^) on the right side. A small fraction of alginate‐rhodamine was added to RGD^1^ to distinguish it from the RGD^0^ compartment. Scale bar = 5 µm. ii) *τ* values from MSCs in gels. *n* = 10 cells. **p < 0.01 via Welch's ANOVA, followed by Dunnett's T3 multiple comparison test. Data are shown as mean ± s.d. B) Quantification of F‐actin in F‐tractin‐tdTomato^+^ MSCs. (i) Representative images showing F‐tractin tdTomato (green) signals in gel‐coated MSCs. Both 2D maximum‐projected and 3D reconstructed images are shown. The dotted line indicates the boundary between the two compartments. Scale bar = 5 µm. ii) Ratio of F‐tractin‐tdTomato mean intensity between left (L) and right (R) compartments. iii) Ratio of tdTomato total intensity between the two compartments. *n* = 18 cells. ***p < 0.001 via Welch's T‐test. C) Quantification of the ratio between the semi‐major axis (a_x_, x: left or right) and the semi‐minor axis (b) of each cell in between two compartments. Each image was taken by reconstructing a confocal z‐stack, orienting the stack so that the boundary plane between the two compartments is vertical, and taking the midplane normal to the boundary plane. (Top) Representative images, scale bar = 5 µm. (Bottom) Quantification from *n* = 15 cells per group. ns: not significant, *p < 0.05, ****p < 0.0001 via Welch's ANOVA, followed by Dunnett's T3 multiple comparison test. D) Finite element analysis showing the distribution of tension (*σ*) within the gel when a cell undergoes non‐uniform isotropic volume expansion with 2.7 µm displacement towards RGD^1^ (left) and 1.6 µm towards RGD^0^ (right).

Cell volume expansion is known to result in increased membrane tension as a function of osmotic pressure and cortical contractility.^[^ ^36^ ^]^ To understand how cell elongation influences membrane tension in response to polarized cell‐matrix interactions, we used a chemical tension reporter that increases *τ* under FLIM in response to higher membrane tension^[^ ^37^ ^]^ (Figure S3A‐i, Supporting Information). MSCs in the microgels show a progressive increase in average membrane tension per cell from symmetric to asymmetric RGD presentation after 1 day in culture (Figure S3A‐ii, Supporting Information), the observation that correlates with faster cell volume expansion kinetics (Figure 3). Membrane tension remains higher on the side of single MSCs in contact with RGD (**Figure** **4**A), suggesting that membrane tension becomes polarized with asymmetric RGD presentation. In contrast, membrane tension is uniform with more symmetric RGD presentation or in bulk gels (Figure S3B‐iii, Supporting Information). Adding a small fraction (1/20) of alginate‐rhodamine to delineate either RGD^1^ or RGD^0^ compartment does not change *τ* values (Figure S3B, Supporting Information). Polarized membrane tension is associated with changes in cytoskeletal structures where the actin cortex in live cells is assembled preferentially towards the RGD‐presenting compartment (Figure 4B). The confocal analysis of the cell membrane relative to each compartment on the midplane shows that MSCs with asymmetric RGD presentation elongate in both directions but the elongation is significantly higher towards the RGD‐presenting compartment (Figure 4C). A model based on finite element analysis shows that non‐uniform isotropic volume expansion is sufficient to recapitulate the experimental results where an MSC (with the radius prior to expansion ≈7.81 µm^[^ ^24^ ^]^) elongates greater towards the RGD side than the RGD‐null side from the center, resulting in a gradient of tension (Figure 4D). Thus, asymmetric cell‐matrix adhesion polarizes membrane tension in single cells during the cell elongation process.

### 2.5 Lineage Specification of Single MSCs by Varying the Spatial Presentation of Integrin Ligands

**Figure 5:**
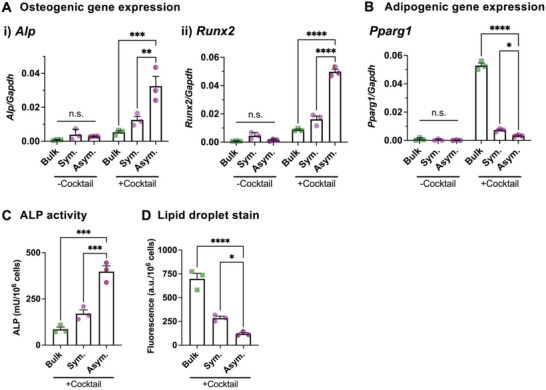
Polarized cell‐matrix interactions drive osteogenic commitment of multipotent MSCs. After encapsulation in gels, MSCs were cultured for 1 day in the basal medium, followed by 10‐day culture in presence of mixed osteogenic and adipogenic cocktails, and subsequent analyses. MSCs in compartmentalized microgels with symmetric (sym.) or asymmetric (asym.) RGD distributions were compared with MSCs in the bulk alginate‐RGD gel with the same elasticity (*E* ≈ 2 kPa). Gene expression of A) osteogenic markers i) *Alp*, ii) *Runx2*, and B) adipogenic marker *Pparg1*. C) Quantification of alkaline phosphatase (ALP) activity. D) Quantification of lipid droplets by a fluorescent neutral lipid stain. *n* = 3 independent experiments. n.s.: not significant, *p < 0.05, **p < 0.01, ***p < 0.001, ****p < 0.0001 via ordinary one‐way ANOVA, followed by Tukey's multiple comparison test. All data are shown as mean ± SD.

Increased cell spreading and generation of traction forces in engineered 3D matrices have generally been linked to the commitment of MSCs toward osteogenic lineages.^[^ ^18^, ^30^ ^]^ We tested whether cell elongation with polarized membrane tension is associated with lineage specification of MSCs in a longer time scale. MSCs in the microgels with symmetric or asymmetric RGD presentation were cultured for 10 days in the absence or the presence of both osteogenesis and adipogenesis‐promoting cocktails. Most MSCs in the microgels with asymmetric RGD presentation do not proliferate (Figure S4A, Supporting Information) and remain viable (Figure S4B, Supporting Information) in this culture condition. These groups were compared with the bulk alginate‐RGD hydrogel (*E* ≈2 kPa). Without the chemical cocktails, no differentiation was observed across all the tested groups. In the presence of the chemical cocktails, MSCs in the microgels with asymmetric RGD presentation show significantly higher gene expression levels of osteogenic markers, including *Alp* and *Runx2* (**Figure** **5**A). In contrast, MSCs in the microgels with symmetric RGD presentation or the bulk gel shows a higher level of an adipogenic marker, *Pparg1* (Figure 5B). Alkaline phosphatase (ALP) enzyme activity assay (Figure 5C) and fluorescent lipid droplet staining (Figure 5D) confirm the osteogenic commitment of multipotent MSCs with asymmetric cell‐matrix adhesion. Confirming the previous results,^[^ ^24^ ^]^ MSCs are committed less towards osteogenic lineages but more towards adipogenic lineages in bulk gels than symmetric microgels due to a higher amount of gel per cell. Thus, the symmetry of cell‐matrix interactions mediates the lineage specification of single stem cells.

### 2.6 Cdc42 is Essential for Single MSCs to Respond to Asymmetric Presentation of Integrin Ligands

**Figure 6:**
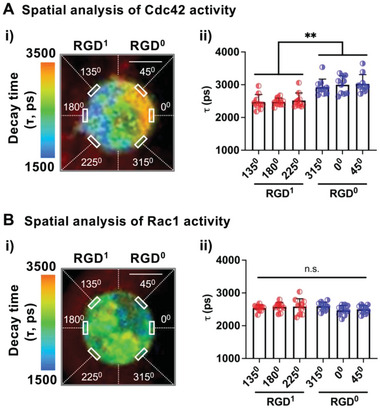
Subcellular localization of Rho GTPase activity with asymmetric cell‐matrix interactions. Measurement of A) Cdc42 activity and B) Rac1 activity at 1 day after encapsulation of MSCs in compartmentalized microgels. Fluorescence lifetime imaging microscopy (FLIM) was used to quantify the decay lifetime (*τ*) of the donor (mTFP1‐WASP CRIB for Cdc42, mTFP1‐PBD for Rac1), which decreases upon FRET due to the interaction with Cdc42‐Venus or Rac1‐Venus. i) Representative image showing *τ* across different regions (angles in counterclockwise directions from 0^0^) of the cell membrane in a microgel consisting of ≈60 µM 1% w/v alginate‐RGD (RGD^1^) on the left side and unmodified RGD (RGD^0^) on the right side. A small fraction of alginate‐rhodamine was added to RGD^1^ to distinguish it from the RGD° compartment. Scale bar = 5 µm. ii) *τ* values from MSCs in gels. *n* = 10 cells. n.s.: not significant, **p < 0.01 via Welch's ANOVA, followed by Dunnett's T3 multiple comparison test. Data are shown as mean ± SD.

To understand what mediates the cellular responses upon asymmetric cell‐matrix interactions, we tested the role of Rho family GTPases, since they are known to be involved in cytoskeleton remodeling and cell polarity.^[^ ^38^ ^]^ To evaluate the activity of Cdc42 and Rac1 in live cells, we introduced FRET‐based biosensors^[^ ^39^ ^]^ to MSCs by nucleofection prior to encapsulation in the gel. FLIM was used to measure *τ* values of interacting monomeric teal fluorescent protein (mTFP1)‐tagged membrane proteins (Wiskott–Aldrich syndrome protein (WASP) Cdc42/Rac interacting binding (CRIB) domain for Cdc42, p21‐activated kinase 1 binding domain (PBD) for Rac1; donor), which are decreased when Venus fluorescent protein‐tagged Cdc42 or Rac1 (acceptor) reach the membrane during the activation process.^[^ ^40^ ^]^ Subcellular analysis shows that *τ* values of the Cdc42 biosensor are significantly lower on the side of single cells that are in contact with RGD (**Figure** **6**A). In contrast, *τ* values of the Rac1 biosensor remain constant across the cell membrane regardless of the presence of RGD (Figure 6B). Thus, Cdc42 activity in single cells becomes polarized in response to asymmetric cell‐matrix adhesion.

**Figure 7:**
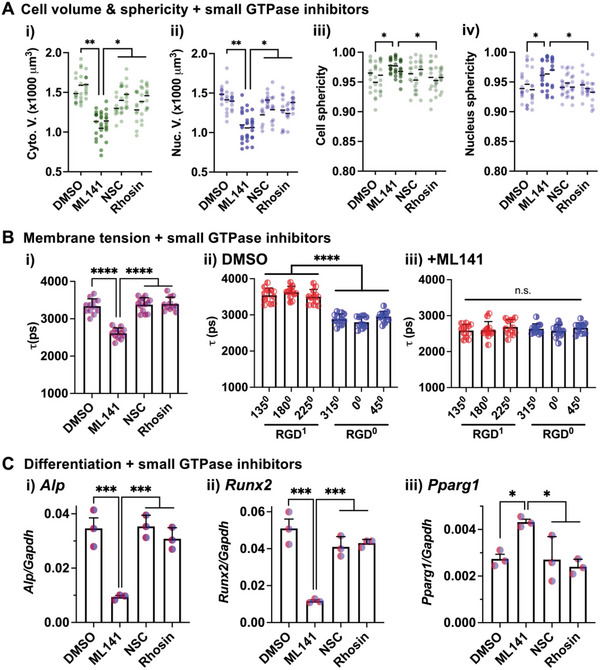
Cdc42 mediates the ability of single MSCs to respond to the asymmetric presentation of integrin ligands. The following inhibitors against small GTPases were tested in gel‐coated MSCs with asymmetric RGD presentation after 1 day in culture in the basal medium: ML141 (10 µM; Cdc42 inhibitor), NSC23766 (NSC; 10 µM; Rac1 inhibitor), Rhosin (30 µM; RhoA inhibitor). DMSO (1:1000 dilution) was used as a negative control. A) Cell volume and sphericity analysis after 2 h drug treatment. i) Cytoplasmic volume, ii) nuclear volume, iii) cell sphericity, and iv) nucleus sphericity. *n* = 3 independent experiments. *p < 0.05, **p < 0.01 via nested one‐way ANOVA, followed by Tukey's multiple comparison test. B) Membrane tension analysis with the lipid tension reporter by FLIM after 2 h drug treatment. i) Mean decay lifetime (*τ*) values per cell. *τ* values across different regions (angles in counterclockwise directions from 0^0^) of the cell membrane after treatment with ii) DMSO and iii) ML141. *n* = 12 cells. n.s.: not significant, ****p < 0.0001 via Welch's ANOVA, followed by Dunnett's T3 multiple comparison test. C) Gene expression analysis after 10‐day culture in the medium supplemented with mixed osteogenic and adipogenic cocktails. The inhibitors were added throughout the culture. Osteogenic markers i) *Alp*, ii) *Runx2*, and adipogenic marker iii) *Pparg1*. *n* = 3 independent experiments. *p < 0.05, ***p < 0.001 via ordinary one‐way ANOVA, followed by Tukey's multiple comparison test. All data are shown as mean ± SD.

To test whether Cdc42 mediates the cellular responses to asymmetric cell‐matrix interactions, we tested the effects of inhibitors against Rho GTPases. After encapsulation of single MSCs in the microgels with asymmetric RGD presentation, they were cultured for 1 day to reach the steady state in cell volume expansion and elongation (Figure 3), followed by 2‐h treatment with inhibitors against Rho GTPases for downstream analyses. ML141 (Cdc42 inhibitor^[^ ^41^ ^]^) but not NSC23766 (Rac1 inhibitor^[^ ^42^ ^]^) and Rhosin (RhoA inhibitor^[^ ^42^ ^]^) reduce both cytoplasmic and nuclear volumes (**Figure** **7**A (i,ii)) while turning cells and nuclei back to the spherical shape (Figure 7A(iii,iv)), suggesting that Cdc42 is required for cell elongation. Importantly, ML141, but not other drugs, reduces overall membrane tension, while equalizing membrane tension across both microgel compartments (Figure 7B). To validate these results with small molecule inhibitors, MSCs were treated with small interfering RNA (siRNA) against Cdc42 or Rac1 prior to encapsulation, which leads to ≈70% decrease in target gene expression (Figure S4C, Supporting Information). Consistently, the knockdown of Cdc42 but not Rac1 reverses the observed changes in cell volume and membrane tension in response to asymmetric RGD presentation (Figure S4D,E, Supporting Information). In addition, ML141 inhibits osteogenic differentiation while promoting adipogenic differentiation (Figure 7C). Together, the results highlight Cdc42 as a unique mediator of cell volume expansion by elongation, the process that is linked to the polarization of membrane tension and lineage specification in response to polarized cell‐matrix interactions.

## 3 Discussion

Our approach takes advantage of laminar flow in microfluidic channels along with selective gelation of alginate‐based droplets containing CaCO_3_‐coated cells to achieve single‐cell encapsulation between two distinct compartments within a microscale gel coating and to obtain a highly purified product simultaneously. The use of high‐viscosity alginate is sufficient to keep the compartments separate when droplets are formed even in the absence of crosslinking. To extend this approach to ionic crosslinking of lower viscosity alginate, for instance, to study the effect of gel stress relaxation,^[^ ^28^ ^]^ it will likely be necessary to implement more rapid crosslinking strategies, such as caged calcium released by light,^[^ ^43^ ^]^ in order to avoid mixing of the compartments. Conversely, it is possible to achieve more complex ligand distribution patterns around single cells by varying ligand concentrations in each compartment, the flow rate ratio between the compartments, or introducing a controlled means to mix the compartments.^[^ ^44^ ^]^

Our results show that MSCs in the microgels expand in volume when RGD is present, while the distribution of RGD around single cells impacts the kinetics and shape of cell volume expansion. It was previously shown that accelerated cell volume expansion in the presence of RGD is a unique phenotype of MSCs encapsulated in microscale hydrogel matrices where material‐to‐cell volume ratios are low, as opposed to bulk gels—this phenotype was shown to be mediated in part by mechanosensitive ion channels,^[^ ^24^ ^]^ which are known to shrink cell volume by water efflux upon activation.^[^ ^45^ ^]^ Since our results show that most MSCs do not proliferate in the microgels with the high molecular weight alginate, it is likely that an initial trigger of cell volume expansion upon RGD binding is osmotically regulated. Indeed, earlier studies showed that cell binding of fibronectin, which contains RGD, triggers osmotic swelling of cells by activating the Na^+^/H^+^ exchanger.^[^ ^46^ ^]^ Cell swelling is rapidly counteracted by regulatory volume decrease^[^ ^24^ ^]^ or requires membrane biosynthesis to support increased membrane tension while preventing cell rupture,^[^ ^47^ ^]^ which could take place during the G1 phase of the cell cycle prior to DNA synthesis. This process will likely be accelerated with faster relaxing microgels made of lower molecular alginate, which were shown to increase cell proliferation.^[^ ^22^ ^]^ Our approach can be used to understand the mechanisms behind the transition from cell volume expansion to biosynthesis as a function of local 3D matrix properties.

Our study shows that the asymmetric distribution of integrin ligands in microgels is sufficient to induce cell polarization in a 3D space where single cells expand in volume by elongation, and membrane tension becomes polarized. Hence, our approach is useful to capture single cells in a specific state of cell polarization. In addition, the results highlight the asymmetric adhesion ligand distribution as an important cue for an early biophysical event that may prime cells to undergo further spreading in 3D hydrogel matrices. Our finite element model shows that cells remain in this primed state when the gel is purely elastic. This model is supported by our experimental results with the high molecular weight alginate, which is known to undergo slower stress relaxation. Given the role of viscoelasticity in promoting 3D cell spreading,^[^ ^28^ ^]^ introducing experimentally testable, time‐dependent variables to our model, including changes in cell volume expansion‐induced strain and membrane tension over time will help explain the transition from the early primed stage to the subsequent cell spreading events. In contrast to the current model, the effective stress on viscoelastic gels is also expected to decrease over time with applied strain from cells due to energy dissipation.

Previous studies showed the importance of cell polarity in driving preferential segregation of cell fate determinants into one of the daughter cells during asymmetric cell division in the mammalian system.^[^ ^6^, ^48^ ^]^ The high molecular weight alginate formulation used in our work is known to keep single cells viable within microgels without proliferation.^[^ ^24^, ^31^ ^]^ Hence, our results provide evidence that cell polarity‐mediated lineage specification does not require cells to divide. In addition, we show that asymmetric cell‐matrix adhesion alone is sufficient to promote the osteogenic differentiation of single MSCs without altering matrix biophysical cues. This mode of lineage specification could potentially be useful in accelerating tissue regeneration where stem cells are primed to undergo differentiation without division in order to rapidly contribute to tissue mass. In contrast, asymmetric cell division is likely necessary to ensure the long‐term maintenance of regenerated tissues by sustaining the stem cell pool. Leveraging degradable^[^ ^18^ ^]^ or lower molecular weight^[^ ^28^ ^]^ formulations, along with approaches to dissect mechanisms of cell division^[^ ^49^ ^]^ will help determine the role of substrate‐directed cell polarity in the asymmetric division of stem cells.

Cdc42 is a known mediator of cell polarity across different species, ranging from yeast^[^ ^50^ ^]^ to mouse hematopoietic stem cells^[^ ^7^ ^]^ by regulating actin assembly.^[^ ^51^ ^]^ In the context of MSCs, an earlier study showed that Cdc42 gene expression is upregulated when MSCs are cultured on a star‐shaped, locally anisotropic 2D substrate where they show polarized cytoskeletons and undergo osteogenesis.^[^ ^10^ ^]^ Cdc42 was also shown to be required for enhanced osteogenic differentiation of MSCs on rough, micro/nano‐textured titanium surfaces.^[^ ^52^ ^]^ Our results suggest that the role of Cdc42 in MSC polarity in response to asymmetric local cell‐matrix interactions is also relevant to 3D microenvironments. While Rac1 was also shown to be upregulated in MSCs on a locally anisotropic 2D substrate,^[^ ^10^ ^]^ our study shows that Rac1 does not play roles in mediating the tested cellular responses upon 3D asymmetric cell‐matrix adhesion. This observation is supported by the previous study showing that Rac1 inhibition does not impact osteogenic differentiation in faster stress‐relaxing 3D hydrogels where cell spreading occurs.^[^ ^28^ ^]^ On 2D substrates, Rac1 mediates the formation of a polarized morphology in rapidly migrating cells, such as neutrophils,^[^ ^53^ ^]^ while Cdc42 regulates integrin‐mediated polarity in slower‐moving or stationary cells.^[^ ^15^, ^54^ ^]^ Our approach provides a platform to test the roles of Rho GTPases in establishing the polarity of different cell types with asymmetric cell‐matrix interactions in a 3D space.

The method can open various avenues of investigation. As a proof‐of‐principle, we show that it is possible to use this approach to tune where RGD is placed within the gel coating around single cells in order to study the biological impact of polarized cell‐matrix interactions. Implementing high‐throughput live imaging approaches along with advanced computational analysis in this context will help reveal more insights in terms of the kinetics and heterogeneity of the cellular responses upon asymmetric cell‐matrix adhesion. Our technology can also be used to study competitive or cooperative effects of different matrix‐bound ligands present in distinct 3D spatial locations on single‐cell functions. In addition, the materials can be further modified to trigger gel degradation or stiffening in each compartment upon external stimuli, such as light,^[^ ^55^ ^]^ enabling temporal tuning of material properties at the subcellular level in a 3D space. Our method can also be adapted to use compartmentalized gel‐coated cells as a building block to direct the assembly of tissues with a finer control of cell‐matrix interactions at the single‐cell level. Since single cells in compartmentalized gel coatings are readily injectable, our approach can provide a practical strategy to deliver single cells in a polarized state ready to be deployed into the host.

## 4 Experimental Section

### Cell Culture

D1 mouse MSCs were purchased from American Type Cell Culture (ATCC, CRL‐12424). D1 MSCs were cultured in complete high‐glucose Dulbecco's modified eagle medium (DMEM, Thermo) supplemented with 1% penicillin‐streptomycin (P/S), 1% GlutaMAX (Thermo), and 10% fetal bovine serum (FBS, Atlanta Biologicals). Cells were cultured until when they reached ≈80% confluence in a 175 cm^2^ flask by detaching them with trypsin‐EDTA (Thermo). D1 MSCs with passage numbers less than 11 were used in this study.

### Alginate Preparation

Sodium alginate (LF200, ≈240 kDa, FMC Biopolymer) was purified by dialyzing against decreasing concentrations of NaCl for 3 days using a dialysis membrane (Spectra/Por 3, 3.5 kDa molecular cut‐off; Repligen), treated with activated charcoal, and sterile filtered, followed by lyophilization. To introduce an integrin adhesion ligand, an Arg–Gly–Asp peptide (GGGGRGDSP; Peptide 2.0) was covalently conjugated to 1% w/v purified alginate in a 2‐morpholinoethanesulfonic acid (MES) buffer (pH 6.5) by 1‐ethyl‐dimethylaminopropyl (EDC) and N‐hydroxysulfosuccinimide (Sulfo‐NHS) (Thermo) with a degree of substitution ≈2 (or ≈0.1% of the total number of carboxylic groups per alginate molecule),^[^ ^56^ ^]^ which was previously shown not to alter *E* of alginate microgels.^[^ ^24^ ^]^ After dialysis, the concentration of RGD conjugated to alginate (≈60 µM at 1% w/v) was verified by the Lavapep Fluorescent Protein and Peptide Quantification Kit (LP‐022010, Gel Company) according to the manufacturer's instructions.

### Microfluidic Device Fabrication

Microfluidic devices were produced by soft lithography as described.^[^ ^57^ ^]^ A negative photoresist SU‐8 25 (Viscosity: 2500 cSt, Kayaku Advanced Materials) was spin‐coated onto a clean silicon wafer (University Wafer) with a defined height, followed by UV exposure through a transparency photomask (CAD/Art Services) for patterning (Figure S1A, Supporting Information). After developing the mold, polydimethylsiloxane (PDMS) (Dow Corning) was mixed with a crosslinker at a ratio of 10:1, degassed, poured, and cured for at least 3 h at 65 °C. The cured PDMS was then peeled off the mold and bonded to an oxygen‐plasma‐treated glass slide for 1 h at 65 °C. Microfluidic channels were treated with Aquapel (PPG Industries) and dried to make them hydrophobic for emulsification. To connect syringes (BD) to microfluidic channels, polyethylene tubing (inner diameter: 0.38 mm; outer diameter 1.09 mm) and 27Gx ½ needed were used. Syringe pumps (Harvard Apparatus) were used to individually control aqueous and oil flow rates.

### Single Cell Encapsulation in Compartmentalized Microgels

CaCO_3_ nanoparticles (CalEssence; 900 nm diameter) were resuspended in a complete DMEM medium and dispersed by sonication with Vibra Cell Sonicator for 1 min at 75% amplitude. The nanoparticles were then centrifuged for 5 min at 50 g to remove aggregated particles, followed by centrifugation for 5 min at 1000 g for collection. Monodispersed CaCO_3_ nanoparticles were resuspended with a serum‐free DMEM medium. The concentration of purified CaCO_3_ nanoparticles was decreased from 24 to 18 mg mL^−1^ with smaller alginate gel coatings with *E* ≈ 2 kPa, and further down to 5 mg mL^−1^ for softer (*E* ≈0.5 kPa) gel coatings. Cells were then incubated with CaCO_3_ by rotation for 1 h at room temperature. Excess CaCO_3_ nanoparticles were washed out by centrifugation for 5 min at 50 g. Three aqueous phases (one middle and two side channels) were prepared with 1% w/v alginate solution in the buffer consisting of DMEM with 50 mM HEPES, 10% FBS, and 1% P/S at pH 7.8. In the middle aqueous phase, CaCO_3_‐coated cells were added. In some experiments, cells were labeled with 2 µM calcein AM (Biotium) to test cell viability during the encapsulation process. In each side aqueous phase, a small amount (final w/v = 0.05%) of 10/60 alginate (≈120 kDa; FMC Biopolymer) conjugated with either Lissamine^TM^ rhodamine B ethylenediamine (Thermo), CF^TM^ 647‐amine (Sigma) or CF^TM^ 350‐amine (Sigma) was added to visualize each gel compartment. Alginate‐RGD was added to either one compartment or both compartments for asymmetric and symmetric RGD presentation, respectively. The oil phase was based on fluorinated oil (HFE‐7500; 3 M) mixed with 3% (for large droplets) or 1% (for small droplets) v/v perfluoropolyether (PFPE, Krytox; Miller Stephenson) as a surfactant, and 0.03% v/v acetic acid as an initiator of Ca^2+^ release from CaCO_3_ nanoparticles. The channel dimensions of the microfluidic device (height × width in µm) were: 70 × 70 and 20 × 30 for large and small droplets, respectively. To focus cells in between two side compartments, the aqueous flow rates were varied in three steps where the side flow rates were progressively increased and the middle flow rate was decreased, while keeping the total aqueous flow rate constant. For large droplets, the total aqueous flow rate was kept at 1.65 µl min^−1^, while the side flow rates varied from 0.2 to 1.55 µl min^−1^ (the middle flow rate from 1.45 to 0.1 µl min^−1^). For small droplets, the total aqueous flow rate was kept at 1 µl min^−1^, while the side flow rates were varied from 0.12 to 0.94 µl min^−1^ (the middle flow rate from 0.88 to 0.06 µl min^−1^). The flow rate of the oil phase was kept 5 times higher than the total aqueous phase. Each step was run for 10 min before moving to the next step. An inverted microscope (Nikon) with a high‐speed camera (Zyla 4.2 sCMOS, Andor) was used to monitor and analyze laminar flow, droplet formation, and cell distribution in the microfluidic device. The emulsion for downstream analyses was collected every 20 min, followed by 20 min rotation at room temperature. The emulsion was then broken by adding 20% 1H,1H,2H,2H‐perfluorooctanol (Alfa Aesar). Gel‐coated cells were washed twice with serum‐free DMEM. Unless otherwise specified, ≈10 000 gel‐coated cells were embedded in 50 µL of 1.25 mg mL^−1^ collagen‐I matrix (Rat tail; Thermo) on a 48‐well glass bottom plate (P48G‐1.5‐6‐F; MatTek), followed by culture at 37 °C in complete DMEM for downstream analyses.

### Cell Encapsulation in Bulk Alginate Hydrogels

Cells were resuspended in 1% w/v alginate‐RGD (≈60 µM) in serum‐free DMEM and rapidly mixed with CaSO_4_ by syringes with a Luer–Lok connector. The final concentration of 10 mM CaSO_4_ was used to form the bulk hydrogel with *E* ≈ 2 kPa.^[^ ^24^, ^31^ ^]^ The mixed solution was deposited between two glass plates with a 1 mm void thickness. After 1.5 h, hydrogels were punched into discs with a 5 mm diameter and cultured on a 96‐well glass bottom plate (P96G‐1.5‐5‐F, MatTek) in complete DMEM.

### Mechanical Characterization of Compartmentalized Gel Coatings Around Single Cells

A glass slide was coated with 0.1 mg mL^−1^ of poly‐_L_‐lysine (Sigma) for 2 h. After washing out poly‐_L_‐lysine, gel‐coated cells were immobilized on the glass slide for 1 h and rinsed with DMEM. The slide was then transferred to an MFP‐3D‐BIO system (Asylum Research) to perform atomic force microscopy with a silicon nitride cantilever with an 18° pyramid tip (MLCT, Bruker). A cantilever spring constant was determined from thermal fluctuations at room temperature (≈40 mN m^−1^) before each analysis. A fluorescent microscope was used to bring the cantilever to the surface of each fluorescently labeled gel compartment. Indentation was then performed under contact mode with 1 µm s^−1^ velocity and force‐distance 500 nm until the trigger deflection voltage (0.5 V) was reached. To calculate Young's modulus (*E*), force–indentation curves were fitted to the Hertzian model with a pyramid indenter and Poisson's ratio (*ν*) = 0.5.

### Confocal Microscopy for 3D Image Analysis

Gel‐coated cells were incubated with 2 µM of calcein AM and 1 µM of Hoechst 33342 (Thermo) for 1 h to stain cytoplasm and nucleus, respectively. Samples were then washed with HBSS and maintained in Fluorobrite DMEM (Thermo) at 37 °C and 5% CO_2_ in the Zeiss LSM 770 confocal microscopy system with a motorized stage and the 20 × /0.8 M27 Plan‐Apochromat objective. To analyze cell volume and sphericity, z‐stacks were captured with 50–80 µm total depth with each image at 0.77 µm for 65–100 images per z‐stack. The stacks were analyzed in Imaris software (Bitplane, version 7.7.2) to perform the 3D reconstruction. Voxels were generated for each gel compartment in red (rhodamine) or blue (CF^TM^ 647), green (calcein), and cyan (Hoechst) signals after automatic thresholding with 10% variation across all the images from different experiments. A gel‐coated cell was considered an outlier if it met one of the following exclusion criteria: 1) It touches another gel‐coated cell, 2) cyan voxels extend beyond the green voxel boundary, 3) green and cyan voxels are not within gel voxels, and 4) gel voxels do not contain green or cyan voxels inside. The total voxels above the threshold were then calculated to quantify the gel, cytoplasmic, and nuclear volumes of each gel‐coated cell. The sphericity of the cell and nucleus was analyzed from the same set of voxels and defined as (*π* ^1/3^(6 *V*)^2/3^)/*A*, where *V* is volume and *A* is surface area. The contact area between the cell and each gel compartment was estimated by the built‐in algorithm (XTension) in Imaris software.

### Inducible Lentiviral Expression of F‐Tractin Fused with Tdtomato

To visualize actin polymers in live cells after encapsulation in gel coatings while minimally impacting baseline cellular functions, the plasmid containing F‐tractin fused with the fluorescent gene tdTomato (a gift from Andrei Karginov, UIC) was cloned into the lentiviral vector pCW57.1 (a gift from David Root, Addgene plasmid #41393) for inducible expression in the presence of doxycycline. F‐tractin is known to selectively bind to polymerized F‐actin.^[^ ^58^ ^]^ Lentiviral particles were produced with a second‐generation lentiviral packaging system (LV003, Applied Biological Materials) using a transfection reagent (Lentifectin, Applied Biological Materials) in HEK293T cells. Lentiviral particles were purified and applied to D1 MSCs at passage 5 with polybrene (8 µg mL^−1^; Sigma) for 3 days, followed by the selection of transduced cells by puromycin (5 µg mL^−1^; Sigma) for 7 days and sorting of tdTomato^+^ cells after doxycycline (500 ng mL^−1^; Cayman Chemical) treatment for 1 day.

### Confocal Analysis of Cell Membrane and F‐Actin in Live Cells

To visualize the cell membrane, MSCs were labeled with 5‐hexadecanoylaminofluorescein (HEDAF, 0.5 mg mL^−1^; Thermo) prior to encapsulation in microgels consisting of red (rhodamine) and blue (CF^TM^ 350) compartments. To visualize F‐actin, F‐tractin‐tdTomato D1 MSCs were treated with doxycycline (500 ng mL^−1^) for 1 day to induce the expression of F‐tractin‐tdTomato prior to encapsulation in microgels consisting of far red (CF^TM^ 647) and blue (CF^TM^ 350) compartments. After 1 day in culture, confocal analysis was done with the 63x/Plan‐Apochromat 1.46NA oil objective at 37 °C and 5% CO_2_. A z‐stack of images was obtained for each cell in a microgel and rotated in Imaris software so that the plane that divided the two gel compartments could be oriented vertically. The horizontal plane perpendicular to the vertical plane was drawn in the middle height of the cell to obtain the midplane. The projected image on the midplane was mapped to each gel compartment and used for subsequent analysis by using ImageJ (2.1.0, National Institutes of Health).

### Measurement of Cell Membrane‐RGD Interactions by Förster Resonance Energy Transfer (FRET) Detected by Fluorescence Lifetime Imaging Microscopy (FLIM)

Cells (4 million mL^−1^) were labeled with the membrane dye HEDAF (0.5 mg mL^−1^) for 1 h in 37 °C as a donor, followed by encapsulation in microgels consisting of red (tetramethylrhodamine (TAMRA)‐conjugated RGD with a degree of substitution ≈2, acceptor) and blue (CF^TM^ 350) compartments. FLIM was then performed to quantify the reduction in donor fluorescence due to acceptor quenching upon FRET (Figure 2A‐i), by using the Ultima Multiphoton Microscope System equipped with a Becker and Hickl time‐correlated single‐photon counting module (Bruker). HEDAF was excited at 820 nm by the Chameleon Ultra II Two‐Photon laser operating at 80 MHz. The emission signal was collected through a 595/60 nm shortpass filter for 30 s. Signal decay time (*τ*) values were extracted by fitting the average photon count versus time graph to a two‐phase exponential decay fit in the data analysis software SPCImage (Becker & Hickl GmbH). The first component of the lifetime in the curve fit was reported in this study since it accounts for the majority of the signal.

### Measurement of Membrane Tension by FLIM

Cells in gels were incubated with 1 µM of Flipper‐TR lipid membrane tension probe (Cytoskeleton, Inc.)^[^ ^37^ ^]^ in complete DMEM for 30 min. One of the gels was labeled with red (rhodamine) fluorescence to distinguish between two compartments. FLIM was performed by exciting the probe at 920 nm, 80 MHz. The emission signal was collected through a 595/50 nm bandpass filter for 1 min, followed by extraction of *τ* values using SPCImage.

### Quantification of Rho GTPase Activity by FRET Detected by FLIM

To quantify Cdc42 or Rac1 activity in live MSCs in gels, MSCs were transfected with pLenti‐Cdc42‐2G (Addgene plasmid #68813) or pLenti‐Rac1‐2G (Addgene plasmid #66111), respectively. Both plasmids were gifts from Olivier Pertz. Each plasmid (4 µg) and 1 million cells were mixed with 100 µl of nucleofector solution from the nucleofector kit (VPE‐1001, Lonza) and electroporated using a high‐viability program (C‐17) in Amaxa (Lonza). Transfected cells were cultured on plastic overnight. After encapsulating transfected cells in microgels and culturing for an additional day, FLIM was performed by exciting the donor (mTFP1‐WASP CRIB for Cdc42, mTFP1‐PBD for Rac1) at 860 nm, 80 MHz. The emission signal was collected through a 595/60 nm shortpass filter for 1 min, followed by extraction of *τ* values using SPCImage.

### Cell Retrieval From Gels

Cells in gels were retrieved by digesting with alginate lyase (4 mg mL^−1^; Sigma), collagenase P (2.5 mg mL^−1^; Sigma), and trypsin‐EDTA (0.125%; Thermo) for 30 min at 37 °C. Samples were then centrifuged at 3000 rpm for 5 min and washed twice with HBSS.

### MSC Differentiation and Assays

MSCs in gels were cultured for 1 day in a basal medium (No. CCM007), followed by the medium supplemented with both osteogenic (No. CCM007) and adipogenic (No. CCM11) cocktails for 10 days. The medium was refreshed on day 5. All reagents for MSC differentiation were purchased from R&D Systems. One‐half of each sample was used to quantify an absolute number of viable cells by calcein staining, while the other half was used to evaluate early osteogenesis or adipogenesis by ALP activity or lipid droplet staining, respectively. To quantify ALP activity, cells were lysed with 200 µl passive buffer (No. E1941, Promega) for 15 min at 4 °C. Each lysate was then added to a black 96‐well plate preloaded with 100 µl 4‐methylumbelliferyl phosphate (4‐MUP) substrate (No. M3168, Sigma). Signals were acquired with excitation at 360 nm and emission at 450 nm using a plate reader. Recombinant mouse ALP protein (Novus Biologicals) was used to generate a standard curve. To quantify lipid droplets in cells, cells were incubated with the LipidSpot™ 610 Lipid Droplet Stain (Biotium) at 37 °C for 30 min. Signals were acquired with excitation at 592 nm and emission at 638 nm using a plate reader.

### Gene Expression Analysis

Cells were lysed with Trizol (500 µL; Thermo) for 10 min. Samples in Trizol were stored at −80 °C for up to one week before processing. Chloroform (100 µL) was added for phase separation. Samples were centrifuged for 10 min at 12500 rpm, 4 °C. The top layer containing RNA was collected into a new tube, and then precipitated with isopropanol (1 mL) for at least 15 min at 4 °C. Samples were then centrifuged at 12500 rpm for 10 min at 4 °C. The supernatant was removed, and the precipitated RNA was washed with 75% ethanol, followed by centrifugation for 5 min at 7500 rpm, 4 °C. After removing ethanol, purified RNA was resuspended in 15 µL of RNase‐free water (Thermo). NanoDrop spectrophotometer (Thermo) was used to quantify RNA concentration and quality. cDNA was obtained by reverse transcription using SuperScript‐III reverse transcriptase (Thermo). For each sample, 50 ng cDNA was added to each well in triplicate, followed by the Power SYBR Green PCR Master Mix (Applied Biosystems). Quantitative PCR was performed in the ViiA7 qPCR system (Thermo). Relative gene expression was calculated using the 2^−ΔCt^ method by normalizing the cycle threshold (Ct) value of each target gene to that of the reference gene (*Gapdh*). Primer sequences are described in **Table** **1**.

**Table 1:** Primer sequences for gene expression studies

*Gapdh*	F: CTTTGTCAAGCTCATTTCCTGG
*(NM_008084.3)*	R: TCTTGCTCAGTGTCCTTGC
*Alp*	F: CTCCAAAAGCTCAACACCAATG
*(NM_007431.3)*	R: ATTTGTCCATCTCCAGCCG
*Runx2*	F: GCTATTAAAGTGACAGTGGACGG
*(NM_009820)*	R: GGCGATCAGAGAACAAACTAGG
*Pparg1*	F: TGTTATGGGTGAAACTCTGGG
*(NM_011146)*	R: AGAGCTGATTCCGAAGTTGG
*Cdc42*	F: CATGTCTCCTGATATCCTACACAAC
*(NM_009861)*	R: TGTCATAATCCTCTTGCCCTG
*Rac1*	F: TGCTTTTCCCTTGTGAGTCC
*(NM_009007)*	R: TCAGCTTCTCAATGGTGTCC

### Chemical Inhibitors

The following chemical inhibitors were purchased from Cayman Chemical: Cilengitide (No. 22289), NSC23766 (No. 13196), and ML141 (No. 18496). Rhosin (No. 5003/10) was purchased from R&D Systems. DMSO was used as a negative control and purchased from Sigma.

### RNA Interference

Small interfering RNAs (siRNAs) were purchased from Horizon Discovery as follows: *Cdc42* (J‐043087‐12‐0002) and *Rac1* (J‐041170‐05‐0002). Scrambled siRNA control (Silencer negative control no. 1) was purchased from Thermo. siRNA (4 nM) was mixed with Lipofectamine RNAiMAX transfection reagent (Thermo) for 20 min in Opti‐MEM (Thermo). The mixture was then applied to cells and cultured for 3 d before cell encapsulation in gels. qPCR was used to confirm the knockdown efficiency of each target gene compared to the scrambled control.

### Finite Element Analysis to Model Gel Stress

A finite element model was implemented using the commercial finite element package Abaqus Standard to solve the linear elastic Eshelby's inclusion problem:^[^ ^59^ ^]^ an expanding cell inclusion inside the gel. The model was formulated as an axisymmetric 3D geometry for its symmetry with respect to the horizontal axis. Both the cell and the gel were defined as simple linear elastic, and incompressible (Poisson's ratio *v* = 0.5) materials. The Young's modulus (*E*) of both the gel and the cell was set to 1500 Pa based on the experimental data (Figure 1F) and previously determined values.^[^ ^24^ ^]^ The model was simulated with a mesh of 13000 elements, in which the convergence was achieved.

To solve this inclusion problem, a non‐uniform isotropic transformation volume expansion (eigenstrain)^[^ ^58^ ^]^ was imposed to simulate cell volume expansion in response to asymmetric ligand (RGD) presentation. Then, the final deformation and stress values of both the cell and the gel were calculated by considering the constraint of the cell expansion due to the gel in finite element analysis. The transformation cell volume expansion was defined based on a quadratic equation which minimizes the expansion of the cell at its center to zero and assigns asymmetric transformation values to the far edges on the horizontal axis. The quadratic distribution of the transformation axial strain as a result of cell volume elongation is expressed as follows

(1) $$\varepsilon_{T}=\left(\frac{\varepsilon_{RGD^{1}}+\varepsilon_{RGD^{0}}}{2R^{2}}\right)X^{2}+\left(\frac{\varepsilon_{RGD^{1}}-\varepsilon_{RGD^{0}}}{2R}\right)X$$

where *R* is the initial radius of an MSC (≈7.81 µm^[^ ^24^ ^]^), *X* is the coordinate along the horizontal axis, with the origin at the center of the undeformed cell with the range between −*R* and *R*, and $\varepsilon_{RGD^{1}}$and $\varepsilon_{RGD^{0}}$ are the maximum transformation strains in the axial direction towards the RGD and RGD‐null sides, respectively.

With $\varepsilon_{RGD^{1}}=0.882$ and $\varepsilon_{RGD^{0}}=0.573$, the maximum horizontal displacement of the cell towards the RGD side is 2.7 µm, the maximum horizontal displacement towards the RGD‐null side is 1.6 µm, and the maximum vertical displacement is 0.65 µm for each of the upper and lower directions. These values closely mirror the experimental results (Figure 4C).

### Statistical Analysis

Statistics were performed as in figure captions using GraphPad Prism version 9.3.1. Unless otherwise noted, statistical comparisons were made by one‐way analysis of variance (ANOVA) followed by Tukey's multiple comparison test when standard deviations did not vary between experimental groups, and Welch's ANOVA, followed by Dunnett's T3 multiple comparison test when standard deviations were variable. For nested datasets, such as cell volume and sphericity of individual cells in response to drugs from each experiment, nested one‐way ANOVA followed by Tukey's test was performed. A *p*‐value less than 0.05 established statistical significance.

## Conflict of Interest

The authors declare no conflict of interest.

## Supporting information

Supporting Information

## Data Availability

The data that support the findings of this study are available from the corresponding author upon reasonable request.
