# Supplementary material for: Deterministic Single Cell Encapsulation in Asymmetric Microenvironments to Direct Cell Polarity
Source: Adv Sci (Weinh). 2022 Dec 1;10(3):2206014. doi: 10.1002/advs.202206014 (PMC9875620; doi:10.1002/advs.202206014)
Supplement: Supplementary file 1 — Supporting Information [file ADVS-10-2206014-s001.pdf]

Supporting Information

Title: Deterministic single cell encapsulation in asymmetric microenvironments to direct cell polarity

Authors: Ik Sung Cho[1,2], Prerak Gupta[1,2], Nima Mostafazadeh[2], Sing Wan Wong[1,2], Saiumamaheswari Saichellappa[1,2], Stephen Lenzini[1,2], Zhangli Peng[2], and Jae-Won Shin[1,2*]

Affiliations: [1]Department of Pharmacology and Regenerative Medicine [2]Department of Biomedical Engineering, University of Illinois at Chicago College of Medicine, Chicago, IL 60612

*Correspondence to J-W. S.: shinjw@uic.edu

This file contains:

Supporting Figures 1-4

Supporting Figures

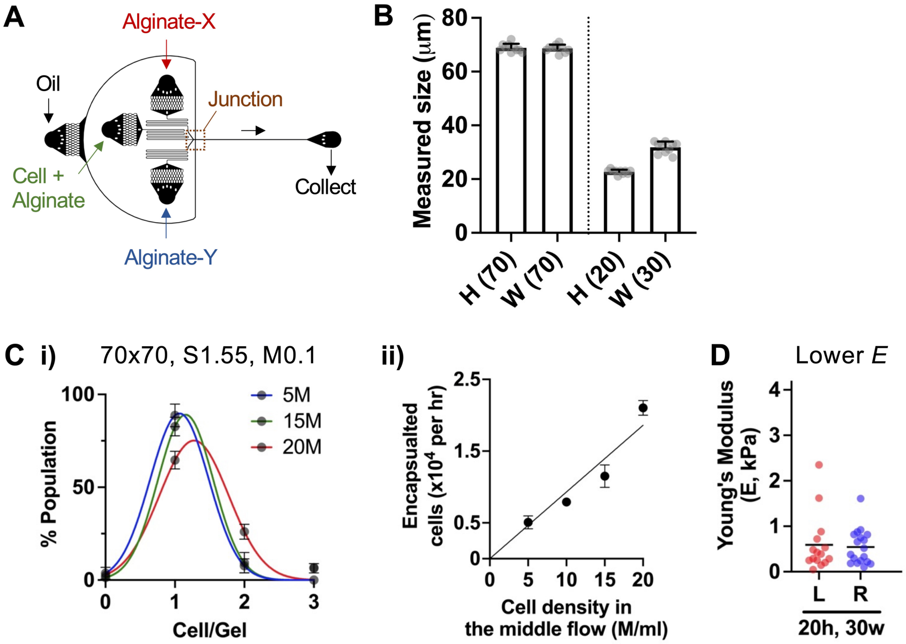

Figure S1. Characterization of the method to encapsulate single cells in compartmentalized microgels. (A) Overview of the microfluidic device. **(B)** Measurement of device height (H) and width (W) after fabrication by microscopy. Values in the round brackets indicate the projected sizes. *n* = 10 devices. **(C)** Quantification of the output from the device. (i) Frequency distribution of gel-coated MSCs with varied cell concentration (per ml) in the middle aqueous phase from the 70h, 70w device with the side aqueous flow = 1.55 μl/min and the middle aqueous flow = 0.1 μl/min. *n* = 3 independent experiments. (ii) The number of gel-coated cells per hour as a function of cell centration in the middle aqueous flow. Data were fitted to $Y = Y_0 \cdot X$, where $Y_0 = 0.00093$. *n* = 3 independent experiments. **(D)** Young's modulus (*E*) of each gel compartment can be tuned down to ~0.5 kPa by lowering CaCO3 concentration to 4 mg/ml as measured by AFM. *n* = 15-18 for each group. All data are shown as mean ± s.d.

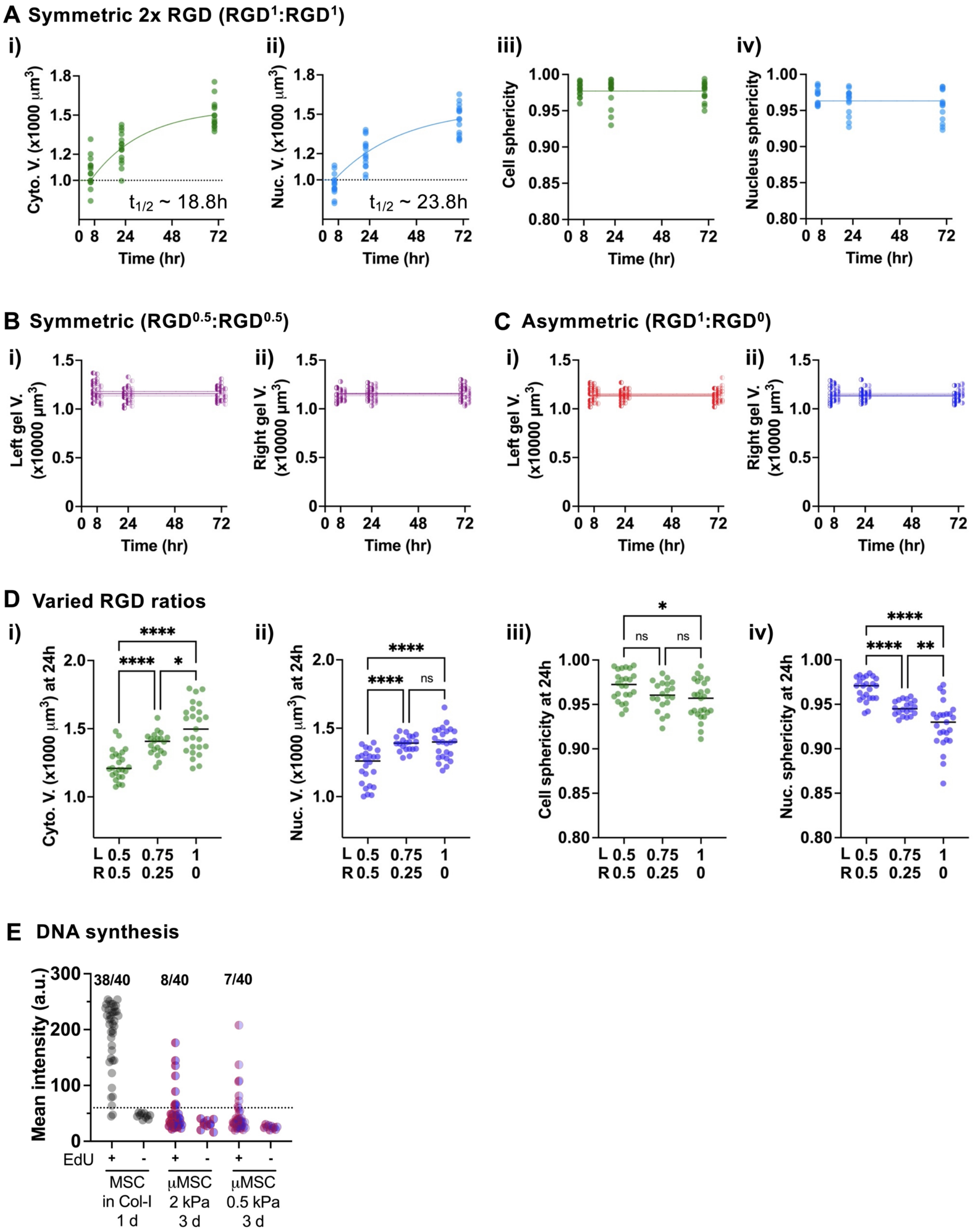

Figure S2. Characterization of single cell volume expansion in compartmentalized microgels. (A) Quantification of (i) cytoplasmic volume, (ii) nuclear volume, (iii) cell sphericity, (iv) nucleus sphericity over 3 days in culture after encapsulation in microgels consisting of 60 µM 1% w/v alginate-RGD (RGD^1) in both compartments. For (i) and (ii), data were fitted to a one-phase association equation starting from t = 8 h: $V = V_0 + (V_m - V_0)(1 - e^{-kt})$, where $V_0 = 1000\ \mu m^3$. For (iii) and (iv), data were fitted to a linear equation with slope = 0. n = 15 cells for each time point. **(B)** Volume of microgels consisting of 30 µM 1% w/v alginate-RGD ($RGD^{0.5}$) in both (i) left and (ii) right compartments. **(C)** Volume of microgels consisting of (i) 60 µM 1% w/v alginate-RGD (RGD^1) in left,

and (ii) no RGD (RGD^0) in right compartments. All data were fitted to a linear equation with slope = 0. n = 3 independent experiments. **(D)** Changes in cell volume and sphericity from symmetric to asymmetric RGD presentation. Quantification of (i) cytoplasmic volume, (ii) nuclear volume, (iii) cell sphericity, (iv) nucleus sphericity of MSCs in microgels after 1 day in culture. The RGD concentration was increased from 30 to 60 μM in the left (L) compartment, and decreased from 30 to 0 μM in the right (R) compartment, while keeping the total alginate w/v at 1% per microgel by adding unconjugated alginate whenever necessary. n = 20-25 cells per group. ns: not significant, *$p < 0.05$, **$p < 0.01$, ****$p < 0.0001$ via Welch's ANOVA, followed by Dunnett's T3 multiple comparisons test. **(E)** Incorporation of EdU by MSCs in collagen-I (Col-I) gel over 1 day in culture or MSCs in microgels with E ~ 2 or 0.5 kPa over 3 days in culture. The dotted line indicates a background signal measured in the absence of EdU. The fluorescence values (in arbitrary units, a.u.) indicate the fractions of cells that incorporated EdU above the background level. n = 40 cells per group.

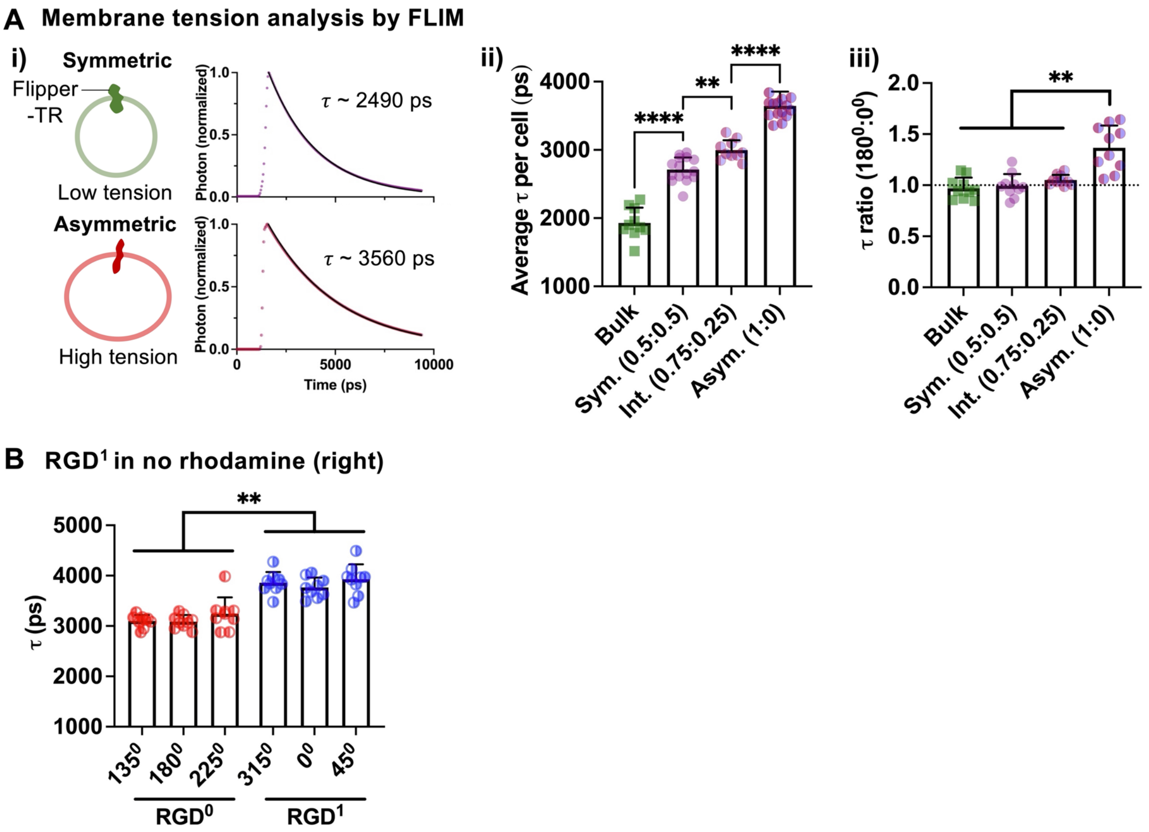

Figure S3. Characterization of cell membrane tension in compartmentalized microgels. (A) Fluorescence lifetime imaging microscopy (FLIM) technique to measure cell membrane tension. (i) After encapsulation in gels, MSCs were labelled with the lipid membrane tension reporter, which undergoes a slower decay in the fluorescence lifetime (τ) when the membrane is under tension due to changes in molecular conformation of the reporter. (ii) Mean τ per cell and (iii) τ ratios between left (180^0) and right (0^0) compartments along the major axis in the bulk gel or in the microgels with varied RGD presentation. $n = 10$ cells per group. **(B)** Spatial analysis of τ values across different regions (angles in counterclockwise directions from 0^0) of the cell membrane in microgels consisting of unmodified RGD (RGD^0) and alginate-rhodamine on the left side and 60 μM 1% w/v alginate-RGD (RGD^1) on the right side. $n = 9$ cells. For all the graphs, $*p < 0.05$, $**p < 0.01$, $****p < 0.0001$ via Welch's ANOVA, followed by Dunnett's T3 multiple comparisons test. All data are shown as mean ± s.d.

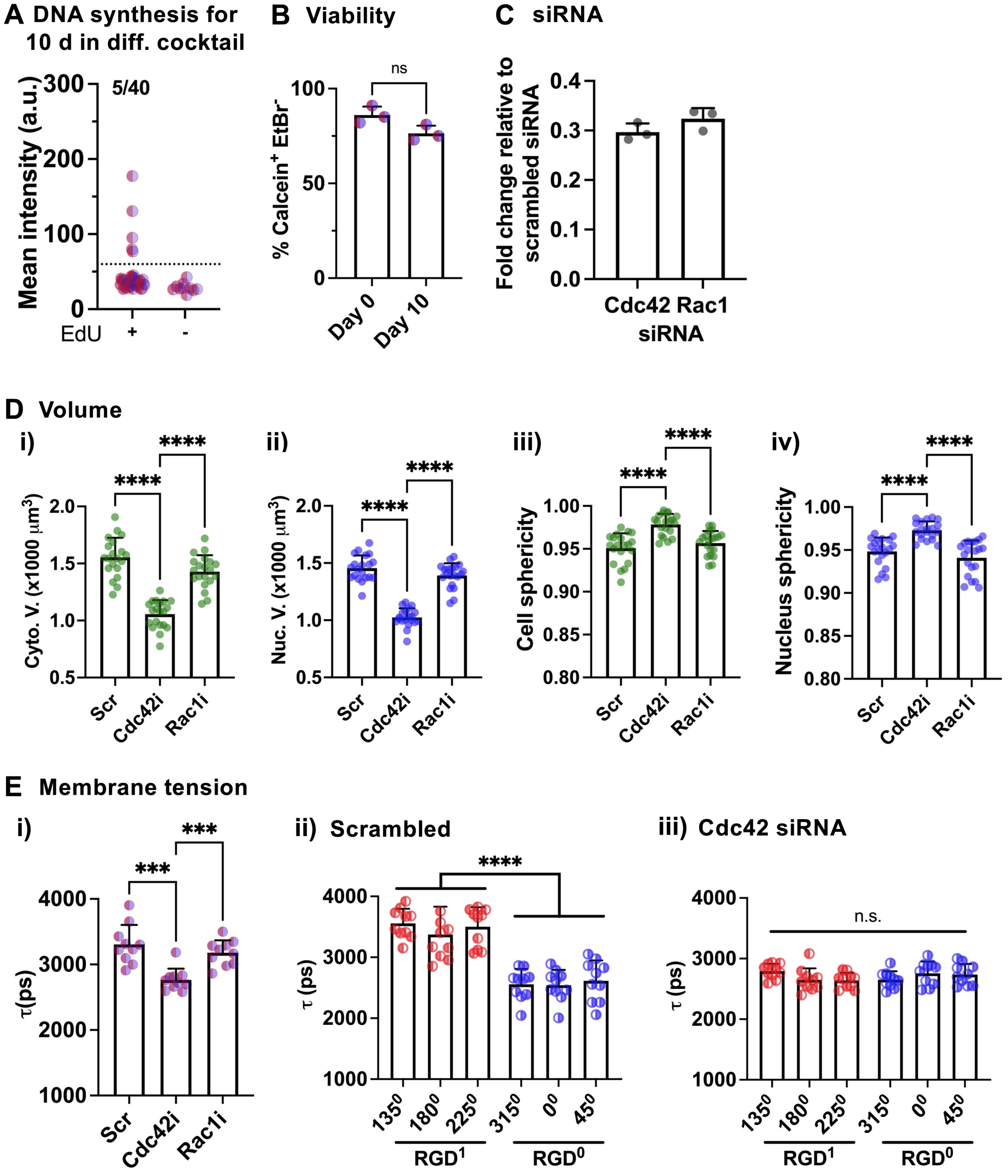

Figure S4. Characterization of the mechanisms behind the cellular phenotypes of MSCs with asymmetric cell-matrix interactions. (A) Incorporation of EdU by MSCs in microgels with asymmetric RGD presentation over 10 days in culture with the differentiation cocktail. The dotted line indicates a background signal measured in the absence of EdU. The fluorescence values (in arbitrary units, a.u.) indicate the fractions of cells that incorporated EdU above the background level. $n = 40$ cells per group. **(B)** Cell viability (percentage of calcein$^+$ ethidium bromide$^-$ cells) right after encapsulation *vs.* after 10 days in culture. $n = 3$ experiments. ns: not significant via unpaired t-test. **(C)** siRNA knockdown efficiency against Cdc42 and Rac1 in MSCs relative to scrambled siRNA measured after 3 days of treatment. $n = 3$ experiments. **(D)** Cell volume and sphericity analysis after siRNA treatment of MSCs, followed by encapsulation and culture for 1 day. (i) Cytoplasmic volume, (ii) Nuclear volume, (iii) Cell sphericity, (iv) Nucleus sphericity. $n = 20$ cells. **(E)** Membrane tension analysis with the lipid tension reporter by FLIM after siRNA treatment. (i) Mean decay lifetime (τ)

values per cell. τ values across different regions (angles in counterclockwise directions from 0^0) of the cell membrane after treatment with (ii) scrambled siRNA and (iii) Cdc42 siRNA. $n = 10$ cells. For **(D)** and **(E),** n.s.: not significant, ***$p < 0.001$, ****$p < 0.0001$ via Welch's ANOVA, followed by Dunnett T3 multiple comparisons test.
